# Written online situational feedback via mobile phone to support self-management of chronic widespread pain: a usability study of a Web-based intervention

**DOI:** 10.1186/1471-2474-12-51

**Published:** 2011-02-25

**Authors:** Ólöf Birna Kristjánsdóttir, Egil A Fors, Erlend Eide, Arnstein Finset, Sandra van Dulmen, Sigrid Hørven Wigers, Hilde Eide

**Affiliations:** 1 Faculty of Nursing, Oslo University College, Oslo, Norway; 2 Division of Psychiatry, St. Olavs Hospital, Trondheim, Norway; 3 Department of Public Health and General Practice, Faculty of Medicine, Norwegian University of Science and Technology, Trondheim, Norway; 4 Stream Networks, Tønsberg, Norway; 5 Department of Behavioral Sciences in Medicine, University of Oslo, Oslo, Norway; 6 NIVEL (Netherlands Institute for Health Services Research), Utrecht, The Netherlands; 7 Jeloy Kurbad Rehabilitation Centre, Moss, Norway; 8 Faculty of Health Science, Buskerud University College, Drammen, Norway

## Abstract

**Background:**

This pretrial study aimed to develop and test the usability of a four-week Internet intervention delivered by a Web-enabled mobile phone to support self-management of chronic widespread pain.

**Methods:**

The intervention included daily online entries and individualized written feedback, grounded in a mindfulness-based cognitive behavioral approach. The participants registered activities, emotions and pain cognitions three times daily using the mobile device. The therapist had immediate access to this information through a secure Web site. The situational information was used to formulate and send a personalized text message to the participant with the aim of stimulating effective self-management of the current situation. Six women participated and evaluated the experience.

**Results:**

The intervention was rated as supportive, meaningful and user-friendly by the majority of the women. The response rate to the daily registration entries was high and technical problems were few.

**Conclusion:**

The results indicate a feasible intervention. Web-applications are fast becoming standard features of mobile phones and interventions of this kind can therefore be more available than before.

**Trial registration number:**

ClinicalTrials.gov: NCT01236209

## Background

Behavior change is an integral part of improved self-management of many chronic health disorders. For people with chronic widespread pain (CWP) or fibromyalgia syndrome (FMS) this is no easy task. The chronic condition of pain without a clear physiological explanation often entails a downward spiral of pain, fear of pain and avoidance behavior, fatigue and depressive symptoms, which makes behavior change extremely challenging [1]. The development of CWP and FMS involves a complex dynamic biopsychosocial process, and multidimensional rehabilitation seems to be the most effective treatment approach [2]. An essential part of the treatment should be an intervention based on cognitive behavioral therapy (CBT) to increase self-management skills [3,4]. CBT with focus on mindfulness and acceptance processes has been found to be effective for people with different chronic health disorders [5], including pain [6]. The goal is for the patient to accept, rather than struggle with unwanted thoughts, emotions and symptoms, and to commit to valued behavior [6,7]. Because of differences in philosophical background and applied techniques between the mindfulness approach and more traditional CBT, some define the mindfulness approach as a new generation of behavioral therapy [8] whereas others view it as consistent with CBT [9].

Internet-administrated cognitive behavioral interventions are increasingly used to support people with health problems [10,11]. Effective operationalization of important elements seems possible because, for some conditions, the effectiveness of Internet-based CBT has been shown to be similar to that of face-to-face CBT [10-12]. A recent meta-analysis of 11 studies shows that Internet-delivered cognitive behavioral interventions for people with chronic pain have a significantly greater effect on pain level than waiting list conditions [13]. The pooled effect size was small, but all the reviewed interventions also improved significantly other health-related and behavioral outcomes (e.g., distress and work capability) compared with the waiting list group.

Results of a recent review of Internet interventions aiming to support behavior changes indicate that interventions including mobile phone text messages and/or some personal online contact can be more helpful in supporting behavior change than Internet interventions without those features [14]. Today's mobile phones commonly include an integrated Internet facility, (e.g., personal digital assistants (PDAs) and smartphones), which opens new possibilities for Internet-based CBT. By using a Web-enabled mobile phone instead of a desktop or laptop computer, the patient can register and send information to the therapist when in different situations. An important goal of CBT is to improve functioning by detecting how automatic thoughts influence feelings and behavior [6]. For this purpose, patients are encouraged to keep some form of record of their thoughts, emotions and behavior (e.g., "What did I feel in the situation?"). The Experience Sampling Method is a way to obtain information on thoughts, feelings and behavior in real time with minimal retrospective bias (e.g., "Right now I am feeling...") [15]. Questions in this format can be answered using the mobile phone and may support self-monitoring [16,17]. The Internet connection makes it possible to submit this information online and make it immediately available to a therapist. The therapist is thereby provided with situational information with a reduced risk of memory bias. Importantly, this also enables the therapist to give the patient prompt feedback on the registered information via a text message [16,18]. In a randomized controlled trial of 76 patients with irritable bowel syndrome, a cluster of symptoms without clear organic abnormalities, the intervention group kept daily online symptom diaries for four weeks and received daily CBT based feedback on a mobile phone. Compared with the control group, the intervention group showed improvements in key symptoms such as catastrophizing thoughts and in quality of life. The effect on catastrophizing was sustained at a three-month follow-up [16]. Using mobile phones and text messaging to support behavior change seems effective for people with different health conditions [14,19]. To our knowledge, no studies exist on the effect of mobile phone interventions to support people with chronic widespread pain [20].

The purpose of this pretrial study was to describe the development and usability of a cognitive behavioral intervention for people with chronic pain using Web-enabled mobile phones. The mobile phones were used to keep online diaries on thoughts, feelings and behavior and to receive situational feedback from a therapist. Acceptability, response rate and user friendliness of the technological system were investigated.

## Methods

### Study sample

This pretrial study was performed from October 2008 to January 2009 on a convenience sample. The goal was to include 6 participants diagnosed with CWP or FMS to test the usability of the intervention. Because women are more often affected by these conditions [4], the choice was made to include only women in this study.

The original aim was to recruit all participants from general practitioners (GPs) but letters and phone calls to the GPs' offices resulted in the recruitment of only two participants. It was therefore decided to contact a rehabilitation center for further recruitment. At the rehabilitation center, the researchers gave information about the study to a group of women with CWP participating in a four-week inpatient multidimensional pain management program including education and pain management in a cognitive setting, various forms of aerobic exercises, stretching, myofascial pain treatment, relaxation and medication as needed (see [21] for details of the program). The first four women to contact the researchers were invited to participate in the study after they had completed the pain management program. Participants received a letter describing the study (either at a visit to their GP's office or at the rehabilitation center). The aims of the study were described as being to develop and implement innovative communication methods to support coping in women with chronic pain. The participants were informed of the intervention's intention to increase awareness of the mind and body relationship and support commitment to valued behavior. Those interested in participating met with the first author and received more information, and signed an informed consent form.

### The intervention

The intervention was developed by building on the experiences of a collaborator (SvD) using similar technology to support people coping with irritable bowel syndrome [16]. For the technological platform, the Open Source Content Management System (Drupal) was used. Data security was maintained through a combination of system design, Hypertext Transfer Protocol Secure (HTTPS) and a proprietary mobile phone authentication system [22]. The content of the intervention (questions in diaries, feedback content and CBT exercises) was chosen by the authors, a multidisciplinary group of health professionals. The theoretical framework was CBT with a focus on mindfulness and acceptance of symptoms. In addition, identifying and working with values and valued activities was emphasized, as recommended by McCracken's mindfulness and acceptance-based CBT for people with chronic pain [6].

### Face-to-face meeting

The intervention started with a one-hour individual meeting between the therapist (a nurse, OBK) and the participant. Each participant was informed about the intervention, asked about her functioning, her goals for health-related behavior and her need for support. They received written exercises to do at home (see Table 1) and an audio CD with relaxation and mindfulness exercises developed in an earlier study [23]. Each participant was lent a Web-enabled mobile phone (HTC TyTN II) with touch screen and keyboard and made the first diary entry at the meeting.

**Table 1:** Key concepts and operationalization of the intervention

Concepts	Diaries	Feedback	Worksheet and CD
Awareness of thought content and feelings, their connection and effect on behavior. Mindfulness.	Fill out entries on thoughts, feelings and behavior three times a day.	Feedback aimed to increase awareness of possible connection between thoughts, feelings and behavior in a recent situation. Mindfulness exercises described and recommended.	Emotion and behavior record [6]. Relaxation and mindfulness exercises (CD, [23]).

Values and valued behavior.	Register planned activities and activities done. Rate satisfaction of today's activity.	Questions and metaphors used to promote reflection on values and valued behavior.	Values and value-based activity. Goals and barriers to value-based living [6]

Acceptance vs. avoidance.	Fill out entries on both positive and challenging thoughts and feelings. Register activity.	Registered information used to give feedback on avoidance behavior or willingness to act in accordance with values despite pain or discouraging thoughts and feelings.	Same as above.

### Online diaries

The participant was asked to complete three diary entries per day using the mobile phone. At a scheduled diary-completion time, she received a Short Message Service (SMS) message with a link to a secure Web site where the diary could be opened and questions answered and submitted back to the server. The morning and evening diary entries were sent at fixed hours chosen by each participant. The second diary entry of the day was sent at a time randomly chosen by the Web server, between 11.30 am and 2 pm. The purpose of including three diary entries, including one at a randomly chosen time, was to encourage self-monitoring of thoughts and feelings at different hours and in different situations. No data were kept on the mobile phone [22]. Two reminder SMS messages were sent within one hour if the diary entry was not returned. If the entry was not submitted within 90 minutes, the form was closed.

The diaries included 19-32 questions. The questions were chosen to support self-monitoring and awareness of feelings, thoughts related to the symptoms and applied self-management strategies. The questions were formulated in line with Experience Sampling Method principles to capture thoughts, feelings and behavior in real time. Most answers were reported by choosing predefined alternatives or scoring on five-point Likert scales. The diaries included questions about current level and interference of pain, planned and achieved activities, feelings, pain-related fear, avoidance, catastrophizing and acceptance (see Table 2). All diaries included a comment field giving participants the opportunity to write a short personal message to the therapist. The participants completed diary entries for a couple of days to get used to the registration, and then they made entries and received daily feedback for four weeks.

**Table 2:** Content and timing of diary entries (* included in)

Content	Morning diary	Midday diary	Evening diary	**Example of questions (Q), statements (S) and answers (A)**.
Pain	x*	x	x	S: Right now my pain level is...
				A: Scale from 0 (no pain) to 10 (worst imaginable pain).
Activity planning	x	x	x	S: In the next couple of hours, I plan to...
				A: List of activities to choose from (including exercise, relaxing and stretching).
Activity evaluation		x	x	Q: How satisfied am I with my level of activity since the last entry?
				A: Five-point Likert scale from "very satisfied" to "very dissatisfied".
Cognitions		x		S: Right now I worry about my pain getting worse.
				A: Five-point Likert scale from "agree totally" to "disagree totally".
Emotions	x		x	S: Right now I am grateful...
				A: Five-point Likert scale from "agree totally" to "disagree totally".
Sleep	x			S: I have slept for...
				A: Less than 2 hours, 2-4 hours, 4-6 hours, 6-8 hours, 8-10 hours, more than 10 hours.
Evaluation of feedback			x	S: The feedback has helped me to stay active.
				A: Yes, No, Unsure.
Open space for comments	x	x	x	

Total number of questions	19	32	24	

### Online written situational feedback

For four weeks, excluding weekends, participants received daily written online feedback within 90 minutes of completing the second (midday) diary entry of the day. The feedback was written by a therapist with a M.Sc. in nursing (OBK). The content of the feedback was supervised by two members of the group (HE, nurse and psychologist with 25 years experience in teaching mindfulness meditation, and EAF, a medical doctor, psychiatrist and a CBT therapist and supervisor). Feedback was sent even if the midday diary was not submitted. The therapist used information from the latest submitted diary. An SMS was sent to signal that feedback was available. The text messages included a link to the Web site where the feedback was posted. There was no limitation on the length of the feedback, which varied from a few sentences to a few paragraphs.

The feedback was intended to suit the participant's situation as reported in the diary. It was written in an empathic communication style and included positive reinforcement, information, metaphors, CBT exercises and questions aiming to encourage mindfulness and willingness to engage in meaningful activities despite pain or other discouraging intrusions (Table 1). Either the instructions for the exercises were written directly in the feedback or participants were referred to the written worksheets or the CD. All participants received feedback texts on values, value-based behavior, mindfulness and acceptance. The texts were tailored to the personal information given at the face-to-face introductory meeting and to the information registered in the diaries. See Table 3 for an example of feedback content.

**Table 3:** Example of a feedback message

Focused breathing
Hi! It seems like you are able to stay active and prioritize pleasurable activities despite your pain. You have already taken a walk and stretched out. Well done! You have just registered that you are not afraid of the pain and that you feel you have some strategies to help you cope with it. You register that your breathing is not relaxed. Can you give yourself a moment to focus on your breathing? Take a deep breath and slowly breathe out a couple of times. I recommend again the focused breathing exercise from last week, the one where you find a quiet spot, comfortable posture, focus on your breathing for a few minutes and--as well as you can--give minimum attention to the content of your thoughts that automatically appear.
Regards, Ann

### Evaluation measures

#### Feasibility of the intervention

To measure feasibility, we developed a questionnaire to measure patients' experiences and satisfaction with the intervention. Participants were also asked to participate in two semi-structured interviews to explore their experience with the intervention (halfway through and after completion). The researchers (OBK and HE) met with the patients individually to evaluate the intervention with questions aiming to capture the experience of participating and suggestions for improvement. Additionally, the experience of the therapist (OBK) was summarized.

#### Subjective usefulness of the feedback

In every evening diary entry, participants were asked about the subjective usefulness of the feedback by two questions with predefined answers to choose from.

#### Assessment questionnaires

To measure possible effects on acceptance and pain-related cognitions, participants were asked to fill out the Chronic Pain Acceptance Questionnaire (CPAQ) [24] and the Pain Catastrophizing Scale (PCS) [25] before and immediately after the intervention period. Catastrophizing and pain acceptance are concepts that seem to mediate effects on treatment outcome in people with chronic pain [26,27]. The CPAQ is a 20-item self-reported instrument containing two subscales: Activity engagement (extent of participation in daily activities despite pain experience) and Pain willingness (willingness to experience pain without trying to control, alter or avoid it). It is scored on a seven-point Likert scale from 0 (never true) to 6 (always true) to give the total score (0-120). Higher scores reflect higher acceptance of pain and higher activities engagement. The PCS is a 13-item questionnaire with three subscales: helplessness, magnification and rumination. Patients rate items on a scale of 0 (not at all) to 4 (all the time). The total score range for PCS is 0-52, with higher scores reflecting higher degrees of catastrophizing. Average pain intensity (previous week) was assessed on a numerical rating scale from 0 (no pain) to 10 (worst possible pain).

#### Treatment fidelity measures

Data were gathered on how many diary entries were submitted by each participant.

### Ethical aspects

The study was approved by the Regional Ethical Committee in Norway and by Norwegian Social Science Services.

### Analyses

Descriptive statistics were calculated as means and frequencies using SPSS version 16. Notes from the interviews were compared and themes identified.

## Results

### Study sample

Six women aged 23-48 years (mean = 36.3) with CWP participated. Four participants were recruited from a rehabilitation center where they had just completed a four-week inpatient multidimensional pain management program. Two were recruited from their GP's office. Three were employed and three were on sick leave. Three were single and the others were cohabiting. Mean average pain level the previous week was 5.33 (SD = 1.51) (0 = no pain, 10 = worst imaginable pain). Table 4 shows pain levels and scores on CPAQ and PCS before and after the intervention period.

**Table 4:** Participants' assessment scores before (T1) and after intervention (T2)

Participant number	Mean pain intensity T1 (0-10)	Mean pain intensity T2 (0-10)	PCS T1 (0-52)	PCS T2 (0-52)	CPAQ T1 (0-120)	CPAQ T2 (0-120)
1 (rehab)	4	5	7	1	83	80
2 (rehab)	7	7.5	18	8	51	64
3 (rehab)	3	5.5	20	25	34	53
4 (GP)	6	6.5	29	24	42	36
5 (GP)	6	Missing	Missing	Missing	Missing	Missing
6 (rehab)	6	6	17	5	65	76

Assessment data were gathered for five participants. Four reported reduced catastrophizing and three reported a higher acceptance of pain after the intervention.

### Evaluation questionnaires

As shown in Table 5, most of the participants perceived participation as supportive, inspiring and meaningful.

**Table 5:** Participants' reports of the intervention

Statement	Agree (n)	Neutral (n)	Disagree (n)
I found the questions understandable	4	1	1
I think I have gained more insight into my complaints	5	1	0
I found it supportive to fill out the diary	5	1	0
I was able to do the given tasks	4	1	1
I have learned some methods I can use to handle my complaints	4	2	0
I found it fun to use the phone	4	1	1
I found it inspiring to use the phone	4	2	0
I found it meaningful to use the phone	5	1	0
I found it time consuming to use the phone	2	2	2
I found it frustrating to use the phone	0	3	3
I found it difficult to use the phone	0	2	4
I found it scary to use the phone	0	1	5
I found it boring to use the phone	0	1	5
I found it shameful to use the phone	0	0	6
I found it disrupted my privacy to use the phone	0	2	4
I found it burdensome to use the phone	1	1	4
I found it disturbing to use the phone	2	2	2
I found it exciting to use the phone	5	1	0
I found the mobile phone user friendly	4	1	1
I found the size of the mobile phone suitable	3	1	2
I found the mobile phone to be too heavy	2	2	2
I found the display screen on the mobile phone good	4	1	1
The letters on the screen were a suitable size	4	1	1
The sound signaling diaries and feedback were suitable	5	0	1

The experience of filling out the diaries was rated as positive and the questions were rated as easily understood. All participants judged the questions in diaries and the content of the feedback to be relevant. Most felt that they were able to do the exercises mentioned in the feedback. All but one participant believed the intervention had increased their insight into their symptoms, and four out of six felt they had learned some new methods to cope with their symptoms. None perceived participation as boring, shameful or invasive of privacy. Most of the participants perceived the mobile phone as user friendly but two found it too heavy and big.

Table 6 shows that most participants agreed that three registrations and one feedback were acceptable per day. Most thought the length of the intervention period to be suitable, but two preferred a longer period. Two women found the number of questions in the diaries to be too many. One participant reported feeling burdened by the intervention, and two agreed that it was somewhat disturbing.

**Table 6:** Evaluation of the intervention structure

	Too many	Suitable	Too few
Number of diary entries per day	1	5	0
Number of questions	3	3	0
Number of feedback messages	0	5	1
Number of weeks receiving feedback	0	4	2

### Subjective usefulness of the feedback

In every evening diary entry, participants were asked to rate the subjective usefulness of the feedback using predefined answers. Fifty percent reported the feedback messages as helpful in maintaining activity (to a level perceived as satisfactory by the participant), and 76% as helpful in staying emotionally well.

### Interviews

The participants appreciated participation in the intervention. They found it useful to fill out the diaries; it increased awareness of their own reactions to the pain and it supported the use of positive coping strategies. The feedback messages were experienced as personal and relevant to the current situation, with a suitable mix of praise, encouragement and CBT exercises. They considered the exercises helpful but some found a few of the exercises difficult to understand. One participant said that she felt she could be more honest when filling out the diaries than she would have been in a face-to-face setting. One of the participants from the rehabilitation center said she felt the intervention had motivated and helped her to integrate what she had learned at the rehabilitation center. It was perceived as supportive in breaking habits and establishing new health behavior. One mentioned that it had made her prioritize reflection on important things. Two women wanted a print out of the feedback messages to have the content available after the intervention.

However, some frustration and difficulties with the intervention were also mentioned. It was occasionally found inconvenient to take the extra mobile phone along, and to fill out the diaries. Some experienced problems with submitting the diaries, e.g. that the registered information disappeared and they had to fill out the diary again. Two mentioned finding it challenging to report on their feelings in the morning diaries. Most wanted a bigger comment field to be able to write more to the therapist. One woman reported sometimes feeling frustrated because she felt misunderstood and was not able to explain herself. The participants' perception of the intervention did not change between the interviews.

The participant who dropped out after three weeks did not experience the intervention as helpful. This woman suffered from flu during the intervention period, something that may have affected her ability and interest in participating.

### Response rate and technical issues

Five patients completed the pilot study. They had a mean response rate of 88% (range 78-94%) to the diaries. One woman recruited from general practice dropped out after three weeks (but still gave her evaluation). Her response rate to filling out the diaries was 42%.

Some experienced a few temporary problems with the Internet connection. It was reported to be frustrating to have filled out a diary form and then not be able to send it because of a validation error. This happened occasionally when the Internet connection was poor, and the validation process from the mobile phone to the server took too long; access was then denied to ensure data security. The researchers were contacted on a few occasions because of problems with submitting a diary form caused by "bad" Internet connections. One feedback message was sent to the wrong participant because the therapist clicked on the wrong feedback button in the system. We had one system breakdown during the pilot period. The system was restored to working order in a few hours and only the submission of the midday entries on one day was disturbed; the evening diary entries that day were completed as usual.

### The therapist's clinical impression

The therapist's experience was mainly positive. She felt that an interactive relationship was established with most of the participants. Nevertheless, the goal of always being empathic and accepting without encouraging avoidant behavior was sometimes found challenging without the ability of providing empathic nonverbal signals and dialog. On occasions, the therapist missed not being able to experience the reaction to questions and statements written in the feedback, especially when metaphors were used to stimulate reflection on values or other exercises anticipated to be emotionally demanding. When a participant reported a high pain score and a low mood it was sometimes tempting to reply with empathy only and not to confront and provide possible challenging questions and/or exercises. The participant's evaluation of how helpful the most recent feedback had been was useful. It might have been beneficial to have the possibility to refer to audio descriptions of more mindfulness exercises (preferably accessible on the mobile phone).

Before writing a feedback message, the therapist would view the three diary entries since the last feedback was sent. It was also necessary to look at the feedback history (all feedback messages were saved on the Web site) to ensure variance in the recommended CBT and mindfulness exercises and to avoid repeating information. No strict feedback template was followed and the amount of time used per feedback varied. Generally, 15-20 minutes were used, with the time decreasing somewhat with experience and the ability to copy and paste content between participants from the growing "bank" of previously written feedback messages.

## Discussion

The aim of this pretrial study was to test the feasibility of delivering an online intervention on a mobile phone and to investigate its acceptability to women with chronic widespread pain and to providers. The intervention was mainly considered user-friendly and helpful, indicating a feasible intervention. The format of the diaries was well accepted and the response rate was generally high (> 80%). This is in accordance with response rates from other studies using electronic diaries [28,29]. Most participants rated the intervention as meaningful and some improvements were shown on measures of catastrophizing and pain acceptance. However, because of the small sample size, these results are of limited value. It should be kept in mind that four out of six women had participated in a rehabilitation program prior to the intervention. It needs to be mentioned that the nurse writing the feedback was also involved in the evaluation interviews and this may have affected the evaluation results. The intervention's framework of mindfulness-based CBT was considered acceptable but further studies are needed to investigate effectiveness and possible therapeutic processes.

Two of the women found some aspects of the intervention disturbing, frustrating and even difficult, e.g. found it challenging to report how they were feeling. Restricted emotional processing and a higher prevalence of alexithymia in patients with FMS than others has been indicated [30]. If and how this could affect the perception and outcome of this intervention needs to be explored.

Recruitment from GPs was not successful and only a few GPs responded to the invitation to include patients in this study. This is not an uncommon experience for e-health interventions [31] or health research in general [32]. Recruitment from the rehabilitation center was more successful. In addition, the experience of the participants from the rehabilitation center was more positive than that of the participants recruited from the GPs. Despite originally intending to recruit only from GPs, it was the impression of both the researchers and the therapist that the intervention might be more accessible following completion of a clinic-based program rather than as an independent intervention. Therapist time per participant was estimated to be about 6-8 hours (including initial meeting and 20 feedback messages). Therapist time is expected to decrease with the construction of a feedback "bank". The predicted economical cost of the intervention is greater than for Web-based interventions with no therapist support. However, results comparing Internet interventions with and without therapist support indicate that the role of an identified therapist has important added value for influencing adherence and outcome [33].

The results from this pretrial study indicate that some possible improvements could be made to the intervention. A few temporary problems with submitting diary entries occurred and should be addressed as they may cause frustration and affect dropout rates. Changes need to be made to eliminate the possibility of sending feedback to the wrong participant. A reduction in the number of items per diary entry might increase acceptability even further [28]. The possibility of participants using their own mobile phones instead of borrowed ones would be preferable. The content of the supplementary audio CD and worksheets could be made available on the participant's phone. Furthermore, to be more consistent with the mindfulness-based CBT framework the audio files should include only mindfulness exercises and not relaxation training. A structured theory-based feedback protocol could make replication of results more feasible, but might compromise the tailored situational aspect of the feedback.

## Conclusions

The results of this pretrial study indicate that written online situational feedback via mobile phone is a feasible intervention for women with chronic widespread pain. The intervention will be further tested in a randomized study to investigate therapeutic effectiveness on behavior change. Web applications are fast becoming standard ingredients in mobile phones and CBT interventions of this kind can be more readily available than before.

## Competing interests

The authors declare that they have no competing interests.

## Authors' contributions

All authors participated in the development of the intervention. OBK recruited participants, helped by SHW who was responsible for Rehabilitation Center patient diagnostics. OBK performed the role of the therapist and participated in evaluation data collection. HE coordinated the study, participated in evaluation interviews and supervised data from participants and feedback. EAF supervised data from participants and feedback. EE was responsible for the design and development of the technological system. OBK and HE analyzed the data and drafted the manuscript. All authors read and approved the final manuscript.

## Pre-publication history

The pre-publication history for this paper can be accessed here:

http://www.biomedcentral.com/1471-2474/12/51/prepub
